# Acyclic nucleoside phosphonates containing the amide bond

**DOI:** 10.1007/s00706-016-1848-x

**Published:** 2016-10-26

**Authors:** Iwona E. Głowacka, Dorota G. Piotrowska, Graciela Andrei, Dominique Schols, Robert Snoeck, Andrzej E. Wróblewski

**Affiliations:** 1 Bioorganic Chemistry Laboratory, Faculty of Pharmacy, Medical University of Lodz, Muszyńskiego 1, 90-151 Lodz, Poland; 2 Rega Institute for Medical Research, KU Leuven, Minderbroedersstraat 10, 3000 Louvain, Belgium

**Keywords:** Nucleotides, Amide bond formation, NMR spectroscopy, Phosphonates

## Abstract

**Abstract:**

To study the influence of a linker rigidity and donor–acceptor properties, the P–CH_2_–**O–CHR**– fragment in acyclic nucleoside phosphonates (e.g., acyclovir, tenofovir) was replaced by the P–CH_2_–**HN–C(O)**– residue. The respective phosphonates were synthesized in good yields by coupling the straight chain of *ω*-aminophosphonates and nucleobase-derived acetic acids with EDC. Based on the ^1^H and ^13^C NMR data, the unrestricted rotation within the methylene and 1,2-ethylidene linkers in phosphonates from series **a** and **b** was confirmed. For phosphonates containing 1,3-propylidene (series **c**) fragments, antiperiplanar disposition of the bulky *O*,*O*-diethylphosphonate and substituted amidomethyl groups was established. The synthesized ANPs P–X–HNC(O)–CH_2_B (X = CH_2_, CH_2_CH_2_, CH_2_CH_2_CH_2_, CH_2_OCH_2_CH_2_) appeared inactive in antiviral assays against a wide variety of DNA and RNA viruses at concentrations up to 100 μM while marginal antiproliferative activity (L1210 cells, IC_50_ = 89 ± 16 μM and HeLa cells, IC_50_ = 194 ± 19 μM) was noticed for the analog derived from (5-fluorouracyl-1-yl)acetic acid and *O*,*O*-diethyl (2-aminoethoxy)methylphosphonate.

**Graphical abstract:**

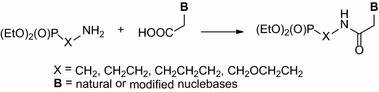

## Introduction

**Fig. 1:**
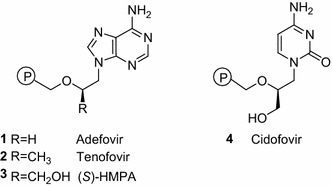
Acyclic nucleotide analogs **1**–**4**

The search for new compounds endowed with antiviral activity has been underway for decades. Several research groups have been active in this field, both in academia and pharmaceutical industry. Despite these efforts, for many viruses efficient drugs have not been yet discovered. In addition, anticancer drugs available so far exhibit limited applicability. The high mutation rate observed for some viruses makes the issue highly complex. Within medications applied to treat viral infections, acyclic nucleoside phosphonates (ANPs) play an important role [1–3]. The prototype of the acyclic nucleoside phosphonates (ANPs), (*S*)-HPMPA (**3**), was never commercialized but it gave rise to three marketed products [cidofovir (**4**), adefovir (**1**), and tenofovir (**2**)] that are often prescribed by physicians. So far known structural modifications of compounds **1**–**4** accomplished within a chain connecting nucleobase and phosphonic acid moieties did not lead to discovery of analogs having higher antiviral activity (Fig. 1).

Analysis of the mechanism of action of ANPs [3, 4] allows to conclude that within structures of newly designed analogs of ANPs, the following fragments have to be installed: (1) a phosphonate unit (P–CH_2_) which prevents enzymatic hydrolysis of the P–O bond present in natural phosphates, while being capable of further phosphorylation; (2) canonical nucleobases or their very close structurally heterocyclic systems to assure efficient interactions with the complementary nucleobases of the other polynucleotide chain; (3) a linker to adjust a distance between a nucleobase and a phosphorus atom which should contain four atoms considered optimal at this moment.

Taking into account high antiviral activity of compounds **1**–**4,** one may be interested in specific interactions of oxygen lone pairs and also of the entire phosphonomethoxy (P–CH_2_–O) fragment. The oxygen atom located within a P–CH_2_–**O**–CHR– fragment can serve as a lone pair donor in intermolecular interactions, and single bonds connecting atoms in the linker ensure free rotation. To modify interactions of atoms located in the linker it is reasonable to consider replacing selected fragments of the linker for another moiety.

During studies on biological activity of peptides, replacements of the amide [–C(O)–NH–] residue for several isosteric fragments (including a methylene ether [–CH_2_–O–] moiety) are commonly applied [5–7]. This particular replacement allows to preserve steric conformity of both bonding systems but essentially influences donor–acceptor interactions and possibilities of a rotation around the specific bonds within the linker [completely free around the CH_2_–O bond (11.3 kJ/mol—barrier to rotation) as compared to the hindered around the C(O)–NH bond (88 kJ/mol—barrier to rotation)]. In general, this isosteric replacement diminishes affinity of modified peptides in comparison with the natural ones.

The proposed structural modification of ANPs **1**–**4** relies on the incorporation of a specific fragment which will be able to increase donor–acceptor interactions within the linker. In our first approach, the [P–CH_2_–**O–CHR**–] fragment will be replaced with the amide [P–CH_2_–**HN–C(O)**–] residue to provide amides **5** (Scheme 1). In addition, a part of the linker containing the amide bond becomes rigid and thus may influence the activity of modified ANPs.

**Figure:**
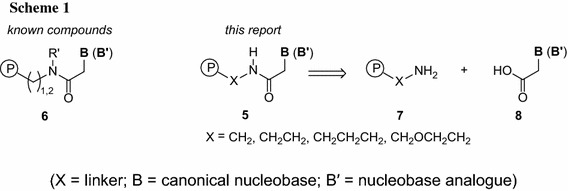

Several decades ago, a concept of phosphonate peptide nucleic acids (PPNA) was introduced [8–11] and a variety of related monomers **6** were synthesized, however, limited to aminomethyl- and aminoethylphosphonate derivatives. Our interest in the synthesis of phosphonates of general formula **5** significantly extends structural diversity of potential new monomers **6** primarily in the aminoalkylphosphonate fragment.

Herein, we wish to describe our studies on the synthesis and the biological activity of the amides **5**. The synthetic strategy involves the formation of the amide bond from the respective acetic acid derivatives **8** and *ω*-aminophosphonates **7** (Scheme 1).

## Results and discussion

To synthesize the first series of the amides **5**, *ω*-aminophosphonates **7a**–**7d** containing straight chain linkers were selected (Scheme 2). The *ω*-aminophosphonates **7a**–**7d** were prepared according to the described procedures [12–14]. Among them, aminomethylphosphonate **7a** was considered as the most important since it was later transformed into the analogs **17a**–**24a** in which nitrogen atoms (N1 or N9) in nucleobases and the phosphorus atom are separated by four bonds, thus providing compounds structurally closest to the drugs **1**–**4**.

**Figure:**
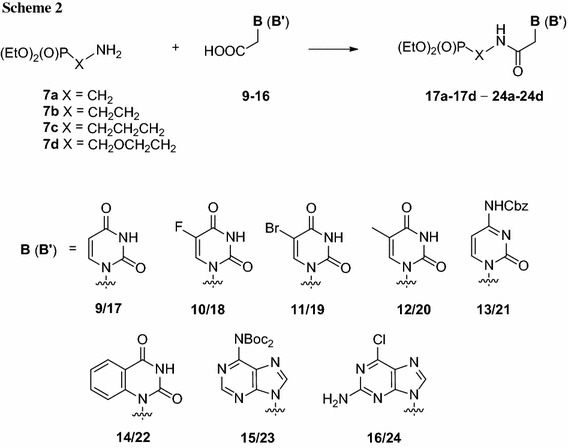

Uracil (**9**)-, 5-fluorouracil (**10**)-, 5-bromouracil (**11**)-, thymine (**12**)-, and benzouracil (**14**)-containing acetic acids are also known compounds and they were synthesized by alkylation of the respective nucleobases by chloro- or bromoacetic acid [15–19].

For future introduction of cytosine- and adenine-acetyl fragments, the protected acids **13** [20] and **15** [21] had to be prepared to significantly increase lipophilicity of the respective amides **21a**–**21d** and **23a**–**23d**. (2-Amino-6-chloropurin-9-yl)acetic acid **16** [22] served as a precursor to guanine-decorated amides **25a**–**25d** (Scheme 3).

**Figure:**
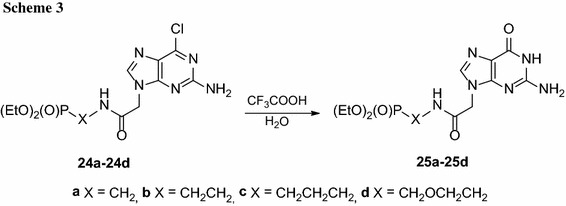

A large number of coupling reagents have been developed for the efficient formation of the amide bond [23, 24] but only some of them proved useful in the peptide chemistry [25]. In search for the coupling system to be applied to our tasks, we were directed by studies of acylation of model secondary amines with (thymine-1-yl)acetic acid (**12**) [26].

When the aminophosphonate **7a** was reacted with the acid **12** in the presence of benzotriazol-1-yloxytris(dimethylamino)phosphonium hexafluorophosphate (BOP) [27], (benzotriazol-1-yloxy)tripyrrolidinophosphonium hexafluorophosphate (PyBOP) [28], *N*-[(dimethylamino)-1*H*-1,2,3-triazolo-[4,5-*b*]pyridin-1-ylmethylene]-*N*-methylmethanaminium hexafluorophosphate *N*-oxide (HATU) [29], and 1-[(1-(cyano-2-ethoxy-2-oxoethylideneaminooxy)-dimethylamino-morpholinomethylene)]methanaminium hexafluorophosphate (COMU) [29] with triethylamine or diethylisopropylamine as additives, the expected amide **20a** could not be isolated from the complex reaction mixtures.

The same coupling was next performed with propylphosphonic anhydride (T3P^®^) [30] and *N*-(3-dimethylaminopropyl)-*N*′-ethylcarbodiimide hydrochloride (EDC) [31, 32] to produce pure amide **20a** [33] in acceptable yields (Table 1). As expected, microwave irradiation significantly accelerated the amide bond formation. In most cases, couplings of the aminophosphonates **7a**–**7d** with nucleobase-substituted acetic acids **9**–**16** in the presence of EDC proceeded almost quantitatively. However, the final products **17a**–**24d** were isolated in moderate yields since several crystallizations were necessary to remove the last traces of triethylamine hydrochloride.

**Table 1:** Optimization of the reaction conditions

Coupling reagent	Additive	Solvent	Time/temp.	Yield/%	Procedure
T3P	TEA	DMF	24 h/r.t.	40	A
EDC	TEA	DMF	72 h/r.t.	60	B
EDC	TEA	CHCl_3_	48 h/r.t.	62	B
EDC	TEA	CHCl_3_	15 min, 1 h/35 °C, 100 W	62	C

The guanine-containing phosphonates **25a**–**25d** were prepared from the 6-chloropurine precursors **24a**–**24d** by hydrolysis in acidic medium (Scheme 3) [34].

### Conformational analysis

**Fig. 2:**
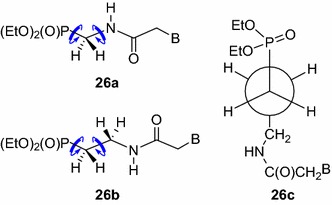
Conformations of phosphonates discussed in this paper

We also aimed to study the conformational behavior of an acyclic linker connecting a phosphorus atom and the amide nitrogen for future structure–activity considerations. Free rotation around P–CH_2_ and H_2_C–NH bonds in phosphonates **17a**–**25a**, as depicted by **26a** in Fig. 2, is evidenced from the vicinal *H*NC*H* couplings of 5.9 Hz observed in the spectra of **21a** and **25a** taken in chloroform-*d* and DMSO-*d*_6_, respectively. In solutions in these solvents as well as in methanol-*d*_4_ *H*_2_CP are equivalent since they always appeared as a one doublet from two-bond HP coupling of 11.8 Hz.

Structure **26b** (Fig. 2) summarizes conformational flexibility of phosphonates **17b**–**25b** which is proved by the observation of the vicinal H_2_C–CH_2_ couplings of 7.6 Hz.

Since ^13^C NMR spectra of phosphonates **17c**–**25c** showed large values (18.1–19.6 Hz) of vicinal PCCC couplings, the antiperiplanar disposition of the *P*–CC–*CH*_*2*_ groups is proposed and represented by the Newman projection **26c**.

Although significant values (11.0–12.1 Hz) were observed in the ^13^C NMR spectra of phosphonates **17d**–**25d** for vicinal PCOC couplings, they cannot be applied with confidence to discuss conformational behavior of the –CH_2_OCH_2_CH_2_– linkage since the angular dependence within a P–C–O–C framework has not yet been established.

### Antiviral activity

All phosphonates **17**–**25** were evaluated for inhibitory activity against a wide variety of DNA and RNA viruses, using the following cell-based assays: (a) human embryonic lung (HEL) cells: herpes simplex virus-1 (KOS strain), herpes simplex virus-2 (G strain), thymidine kinase-deficient (acyclovir resistant) herpes simplex virus-1 (TK^−^ KOS ACV^r^ strain), vaccinia virus, adenovirus-2, vesicular stomatitis virus, cytomegalovirus (AD-169 strain and Davis strain), varicella-zoster virus (TK^+^ VZV strain and TK^−^ VZV strain); (b) HeLa cell cultures: vesicular stomatitis virus, Coxsackie virus B4 and respiratory syncytial virus; (c) Vero cell cultures: parainfluenza-3 virus, reovirus-1, Sindbis virus, Coxsackie virus B4, Punta Toro virus, yellow fever virus; (d) CrFK cell cultures: feline corona virus (FIPV) and feline herpes virus (FHV); (e) MDCK cell cultures: influenza A virus (H1N1 and H3N2 subtypes) and influenza B virus. Ganciclovir, cidofovir, acyclovir, brivudine, zalcitabine, zanamivir, alovudine, amantadine, rimantadine, ribavirin, dextran sulfate (molecular weight 10,000, DS-10000), mycophenolic acid, Hippeastrum hybrid agglutinin (HHA) and Urtica dioica agglutinin (UDA) were used as the reference compounds. The antiviral activity was expressed as the EC_50_: the compound concentration required to reduce virus plaque formation (VZV) by 50% or to reduce virus-induced cytopathogenicity by 50% (other viruses). None of the tested compounds showed appreciable antiviral activity toward any of the tested DNA and RNA viruses at concentrations up to 100 μM, nor affected cell morphology of HEL, HeLa, Vero, MDCL, and CrFK cells.

### Cytostatic activity

The 50% cytostatic inhibitory concentration (IC_50_) causing a 50% decrease in cell proliferation was determined against murine leukemia L1210, human lymphocyte CEM, human cervix carcinoma HeLa, and human dermal microvascular endothelial cells (HMEC-1).

**Fig. 3:**
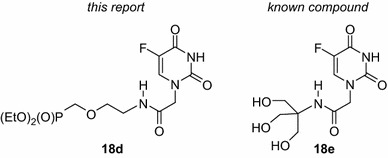
Active derivatives of 5-fluorouracil

Among all tested compounds, only phosphonate **18d** showed slight antiproliferative activity toward L1210 (IC_50_ = 89 ± 16 μM) and HeLa cells (IC_50_ = 194 ± 19 μM) and thus enlarges a collection of 5-fluorouracil derivatives which together with the parent compound have been widely clinically applied in therapies of various cancers [35]. Furthermore, the closest structural analog of **18d** (compound **18e**) was found active in vitro against L1210 cells at concentration of 15 μM [36] (Fig. 3).

## Conclusion

Replacement of the P–CH_2_–**O–CHR**– fragment in ANPs (e.g., acyclovir, tenofovir) by the P–CH_2_–**HN–C(O)**– residue was introduced to study the influence of a linker rigidity and changes in donor–acceptor properties. To elaborate an appropriate synthetic methodology, a series of the 36 respective phosphonates as *O*,*O*-diethyl esters was prepared in good yields by the EDC-induced coupling of the straight chain *ω*-aminophosphonates and nucleobase-derived acetic acids. Besides the rigidity of the amide bond based on the ^1^H and ^13^C NMR data, it was concluded that the unrestricted rotation within the methylene (series **a**) and 1,2-ethylidene (series **b**) linkers takes place while for phosphonates containing 1,3-propylidene (series **c**) fragments antiperiplanar disposition of the bulky *O*,*O*-diethylphosphonate and substituted amidomethyl groups was observed. The phosphonates **17a**–**25d** appeared inactive in antiviral assays against a wide variety of DNA and RNA viruses at concentrations up to 100 μM. Marginal antiproliferative activity (L1210, IC_50_ = 89 ± 16 μM and HeLa, IC_50_ = 194 ± 19 μM) was noticed for the phosphonate **18d** derived from (5-fluorouracyl-1-yl)acetic acid and *O*,*O*-diethyl (2-aminoethoxy)methylphosphonate. Studies on the analogous phosphonates containing functionalized linkages are currently ongoing in our laboratory and the most active diethyl esters will be transformed into the free ANP and further derivatized to selected prodrug phosphonates [37].

## Experimental

^1^H NMR spectra were recorded in CD_3_OD, CDCl_3_, or DMSO-*d*_6_ on the following spectrometers: Varian Gemini 2000BB (200 MHz) and Bruker Avance III (600 MHz) with TMS as internal standard. ^13^C NMR spectra were recorder for CD_3_OD, CDCl_3_, or DMSO-*d*_6_ solution on the Bruker Avance III at 151.0 MHz. ^31^P NMR spectra were performed on the Varian Gemini 2000BB at 81.0 MHz or on Bruker Avance III at 243.0 MHz. IR spectral data were measured on a Bruker Alpha-T FT-IR spectrometer. Melting points were determined on a Boetius apparatus. Elemental analyses were performed by Microanalytical Laboratory of this Faculty on Perkin Elmer PE 2400 CHNS analyzer and their results were found to be in good agreement (±0.3%) with the calculated values.

The following absorbents were used: column chromatography, Merck silica gel 60 (70–230 mesh); analytical TLC, Merck TLC plastic sheets silica gel 60 F_254_. TLC plates were developed in chloroform–methanol solvent systems. Visualization of spots was effected with iodine vapors. All solvents were purified by methods described in the literature.

### Synthesis of amides 17–24

#### General procedure A

A solution of 0.100 g aminophosphonate **7a** (0.598 mmol) in 2 cm^3^ DMF containing 0.100 g (thymine-1-yl)acetic acid (**12**, 0.544 mmol) was cooled to 0 °C and 0.152 cm^3^ TEA (1.09 mmol) followed by a 0.481 cm^3^ of a 50% solution of T3P^®^ in DMF (0.816 mmol) were added. The reaction mixture was stirred at room temperature for 24 h, concentrated in vacuo and treated with 5 cm^3^ water. A solid was filtered off, air dried, and subjected to chromatography on a silica gel column with chloroform–methanol mixtures (20:1, 10:1 v/v) to give pure compound **20a** (0.072 g, 40% yield) as a white powder.

#### General procedure B

To a solution of aminophosphonates **7a**–**7d** (1.00 mmol) in 2 cm^3^ DMF or chloroform the respective acetic acids **9**–**16** (1.00 mmol), EDC × HCl (1.00 mmol), and TEA (1.00 mmol) were added. The reaction mixture was stirred at room temperature for 48–72 h and then concentrated in vacuo. The residue was chromatographed on a silica gel column with chloroform–methanol mixtures and crystallized from the appropriate solvents.

#### General procedure C

To a solution of aminophosphonates **7a**–**7d** (1.00 mmol) in 2 cm^3^ chloroform the respective acetic acids **9**–**16** (1.00 mmol), EDC × HCl (1.00 mmol), and TEA (1.00 mmol) were added. The reaction mixture was irradiated (100 W) at 35 °C for the specified time (15 min–3 h) and then concentrated in vacuo. The residue was chromatographed on a silica gel column with chloroform–methanol mixtures and crystallized from the appropriate solvents.

##### *Diethyl [2*-*(3,4*-*dihydro*-*2,4*-*dioxopyrimidin*-*1(2H)*-*yl)acetamido]methylphosphonate* (**17a**, C_11_H_18_N_3_O_6_P)

The crude product obtained from 0.100 g diethyl 2-aminomethylphosphonate (**7a**, 0.60 mmol) and 0.097 g (uracil-1-yl)acetic acid (**9**, 0.60 mmol) in the presence of 0.115 g EDC × HCl (0.60 mmol) and 0.083 cm^3^ TEA (0.60 mmol) according to the general procedure B was chromatographed with chloroform–methanol mixtures (50:1, 20:1 v/v) to give pure compound **17a** (0.106 g, 55% yield) as a white powder. M.p.: 215–217 °C; IR (KBr): $\bar{\nu }$ = 3254, 3058, 2997, 2882, 2831, 1703, 1679, 1259, 1193, 1031 cm^−1^; ^1^H NMR (200 MHz, CD_3_OD): *δ* = 7.56 (d, ^3^ *J* = 7.9 Hz, 1H, HC6), 5.71 (d, ^3^ *J* = 7.9 Hz, 1H, HC5), 4.51 (d, ^3^ *J* = 1.4 Hz, 2H, C(O)CH_2_), 4.26–4.11 (m, 4H, 2 × POC*H*_2_CH_3_), 3.78 (d, ^2^ *J* = 11.8 Hz, 2H, PCH_2_N), 1.37 (t, *J* = 7.0 Hz, 6H, 2 × POCH_2_C*H*_3_) ppm; ^13^C NMR (151 MHz, CD_3_OD): *δ* = 167.92 (d, *J* = 3.7 Hz), 165.38, 151.36, 146.37, 100.85, 62.79 (d, *J* = 6.5 Hz, POC), 49.56, 34.23 (d, *J* = 158.5 Hz, PC), 15.28 (d, *J* = 5.8 Hz, POC*C*) ppm; ^31^P NMR (243 MHz, CD_3_OD): *δ* = 23.6 ppm.

##### *Diethyl 2*-*[2*-*(3,4*-*dihydro*-*2,4*-*dioxopyrimidin*-*1(2H)*-*yl)acetamido]ethylphosphonate* (**17b**, C_12_H_20_N_3_O_6_P)

The crude product obtained from 0.100 g diethyl 2-aminoethylphosphonate (**7b**, 0.550 mmol) and 0.094 g (uracil-1-yl)acetic acid (**9**, 0.55 mmol) in the presence of 0.105 g EDC × HCl (0.550 mmol) and 0.077 cm^3^ TEA (0.55 mmol) according to the general procedure B was chromatographed with chloroform–methanol mixtures (50:1, 20:1 v/v) to give pure compound **17b** (0.095 g, 52% yield) as a white powder. M.p.: 138–140 °C; IR (KBr): $\bar{\nu }$ = 3340, 2986, 2953, 2896, 2826, 1696, 1673, 1236, 1024 cm^−1^; ^1^H NMR (600 MHz, CD_3_OD): *δ* = 7.52 (d, ^3^ *J* = 7.9 Hz, 1H, HC6), 5.70 (d, ^3^ *J* = 7.9 Hz, 1H, HC5), 4.43 (s, 2H, C(O)CH_2_), 4.18–4.10 (m, 4H, 2 × POC*H*_2_CH_3_), 3.48 (dt, *J* = 12.3 Hz, *J* = 7.6 Hz, 2H, PCH_2_C*H*_2_), 2.10 (dt, *J* = 15.3 Hz, *J* = 7.6 Hz, 2H, PC*H*_2_), 1.36 (t, *J* = 7.1 Hz, 6H, 2 × POCH_2_C*H*_3_) ppm; ^13^C NMR (151 MHz, CD_3_OD): *δ* = 167.92, 165.38, 151.49, 146.38, 100.95, 62.09 (d, *J* = 6.5 Hz, POC), 49.81, 33.41, 24.87 (d, *J* = 139.0 Hz, PC), 15.31 (d, *J* = 6.2 Hz, POC*C*) ppm; ^31^P NMR (243 MHz, CD_3_OD): *δ* = 29.34 ppm.

##### *Diethyl 3*-*[2*-*(3,4*-*dihydro*-*2,4*-*dioxopyrimidin*-*1(2H)*-*yl)acetamido]propylphosphonate* (**17c**, C_13_H_22_N_3_O_6_P × H_2_O)

The crude product obtained from 0.080 g diethyl 3-aminopropylphosphonate (**7c**, 0.41 mmol) and 0.070 g (uracil-1-yl)acetic acid (**9**, 0.41 mmol) in the presence of 0.079 g EDC × HCl (0.41 mmol) and 0.057 cm^3^ TEA (0.41 mmol) according to the general procedure B was chromatographed with chloroform–methanol mixtures (50:1, 20:1 v/v) and the selected fractions were crystallized from ethyl acetate to give pure compound **17c** (0.046 g, 32% yield) as a white solid. M.p.: 120–122 °C; IR (KBr): $\bar{\nu }$ = 3327, 3101, 2989, 2937, 2885, 2835, 1697, 1670, 1563, 1240, 1039 cm^−1^; ^1^H NMR (200 MHz, CD_3_OD): *δ* = 7.56 (d, ^3^ *J* = 7.9 Hz, 1H, HC6), 5.70 (d, ^3^ *J* = 7.9 Hz, 1H, HC5), 4.45 (s, 2H, C(O)CH_2_), 4.28–4.03 (m, 4H, 2 × POC*H*_2_CH_3_), 3.30 (t, *J* = 6.9 Hz, 2H, PCH_2_CH_2_C*H*_2_), 1.95–1.68 (m, 4H, PC*H*_2_C*H*_2_), 1.36 (t, *J* = 7.1 Hz, 6H, 2 × POCH_2_C*H*_3_) ppm; ^13^C NMR (151 MHz, CD_3_OD): *δ* = 168.08, 165.41, 151.44, 146.46, 100.85, 61.90 (d, *J* = 6.0 Hz, POC), 50.00, 39.34 (d, *J* = 18.8 Hz, PCC*C*), 22.13 (d, *J* = 4.5 Hz, PC*C*), 21.91 (d, *J* = 142.1 Hz, PC), 15.30 (d, *J* = 5.7 Hz, POC*C*) ppm; ^31^P NMR (81 MHz, CD_3_OD): *δ* = 33.69 ppm.

##### *Diethyl [2*-*[2*-*(3,4*-*dihydro*-*2,4*-*dioxopyrimidin*-*1(2H)*-*yl)acetamido]ethoxy]methyl phosphonate* (**17d**, C_13_H_22_N_3_O_7_P)

The crude product obtained from 0.050 g diethyl (2-aminoethoxy)methylphosphonate (**7d**, 0.237 mmol) and 0.040 g (uracil-1-yl)acetic acid (**9**, 0.237 mmol) in the presence of 0.045 g EDC × HCl (0.237 mmol) and 0.033 cm^3^ TEA (0.237 mmol) according to the general procedure B was chromatographed with chloroform–methanol mixtures (50:1, 20:1 v/v) and the selected fractions were crystallized from ethyl acetate–hexane mixture to give pure compound **17d** (0.060 g, 70% yield) as white needles. M.p.: 116–117 °C; IR (KBr): $\bar{\nu }$ = 3320, 3098, 2984, 2980, 2951, 2888, 2821, 1695, 1673, 1257, 1031 cm^−1^; ^1^H NMR (200 MHz, CD_3_OD): *δ* = 7.56 (d, ^3^ *J* = 7.9 Hz, 1H, HC6), 5.70 (d, ^3^ *J* = 7.9 Hz, 1H, HC5), 4.48 (s, 2H, C(O)CH_2_), 4.29–4.14 (m, 4H, 2 × POC*H*_2_CH_3_), 3.92 (d, *J* = 8.5 Hz, 2H, PCH_2_O), 3.69 (t, *J* = 5.3 Hz, 2H, PCH_2_OC*H*_2_CH_2_), 3.46 (t, *J* = 5.3 Hz, 2H, PCH_2_OCH_2_C*H*_2_), 1.38 (t, *J* = 7.0 Hz, 6H, 2 × POCH_2_C*H*_3_) ppm; ^13^C NMR (151 MHz, CD_3_OD): *δ* = 168.04, 165.39, 151.44, 146.44, 100.83, 71.44 (d, *J* = 12.1 Hz, PCO*C*), 64.19 (d, *J* = 166.9 Hz, PC), 62.77 (d, *J* = 6.6 Hz, PO*C*), 49.70, 38.96, 15.36 (d, *J* = 5.5 Hz, POC*C*) ppm; ^31^P NMR (81 MHz, CD_3_OD): *δ* = 23.10 ppm.

##### *Diethyl [2*-*(5*-*fluoro*-*3,4*-*dihydro*-*2,4*-*dioxopyrimidin*-*1(2H)*-*yl)acetamido]methylphosphonate* (**18a**, C_11_H_17_FN_3_O_6_P × 0.5H_2_O)

The crude product obtained from 0.107 g diethyl aminomethylphosphonate (**7a**, 0.640 mmol) and 0.120 g (5-fluorouracil-1-yl)acetic acid (**10**, 0.640 mmol) in the presence of 0.123 g EDC × HCl (0.640 mmol) and 0.089 cm^3^ TEA (0.64 mmol) according to the general procedure B was filtered and crystallized from a methanol–diethyl ether mixture to give pure compound **18a** (0.127 g, 59% yield) as a white powder. M.p.: 205–208 °C; IR (KBr): $\bar{\nu }$ = 3224, 3061, 3000, 2941, 2830, 1738, 1700, 1034 cm^−1^; ^1^H NMR (600 MHz, CD_3_OD): *δ* = 7.80 (d, *J* = 6.2 Hz, 1H, HC6), 4.46 (s, 2H, C(O)CH_2_), 4.20–4.15 (m, 4H, 2 × POC*H*_2_CH_3_), 3.76 (d, *J* = 11.8 Hz, 2H, PCH_2_), 1.36 (t, *J* = 7.1 Hz, 6H, 2 × POCH_2_C*H*_3_) ppm; ^13^C NMR (151 MHz, CD_3_OD): *δ* = 167.78 (d, ^3^ *J* = 3.2 Hz, *C*(O)NHCP), 158.54 (d, *J* = 26.2 Hz), 150.12, 140.17 (d, *J* = 232.5 Hz), 130.30 (d, *J* = 34.1 Hz), 62.82 (d, *J* = 6.0 Hz, POC), 49.56, 34.27 (d, *J* = 158.6 Hz, PC), 15.28 (d, *J* = 5.8 Hz, POC*C*) ppm; ^31^P NMR (273 MHz, CD_3_OD): *δ* = 22.71 ppm.

##### *Diethyl 2*-*[2*-*(5*-*fluoro*-*3,4*-*dihydro*-*2,4*-*dioxopyrimidin*-*1(2H)*-*yl)acetamido]ethylphosphonate* (**18b**, C_12_H_19_FN_3_O_6_P)

The crude product obtained from 0.114 g diethyl 2-aminoethylphosphonate (**7b**, 0.629 mmol) and 0.118 g (5-fluorouracil-1-yl)acetic acid (**10**, 0.629 mmol) in the presence of 0.121 g EDC × HCl (0.629 mmol) and 0.088 cm^3^ TEA (0.63 mmol) according to the general procedure B was chromatographed with chloroform–methanol mixtures (100:1, 50:1, 20:1 v/v) and the selected fractions were crystallized from a methanol–diethyl ether mixture to give pure compound **18b** (0.131 g, 59% yield) as a white solid. M.p.: 185–186 °C; IR (KBr): $\bar{\nu }$ = 3241, 3063, 3035, 2982, 2930, 1742, 1657, 1630, 1215, 1056 cm^−1^; ^1^H NMR (600 MHz, CD_3_OD): *δ* = 7.80 (d, *J* = 6.2 Hz, 1H, HC6), 4.41 (s, 2H, C(O)CH_2_), 4.19–4.10 (m, 4H, 2 × POC*H*_2_CH_3_), 3.51-3.46 (m, 2H, PCH_2_C*H*_2_), 2.13–2.08 (m, 2H, 2H, PC*H*_2_), 1.36 (t, *J* = 7.1 Hz, 3H, POCH_2_C*H*_3_) ppm; ^13^C NMR (151 MHz, CD_3_OD): *δ* = 167.76, 158.53 (d, *J* = 25.6 Hz), 150.19, 140.24 (d, *J* = 231.0 Hz), 130.27 (d, *J* = 34.1 Hz), 62.07 (d, *J* = 6.6 Hz, POC), 49.78, 33.40 (d, *J* = 1.6 Hz), 24.86 (d, *J* = 139.6 Hz, PC), 15.28 (d, *J* = 5.7 Hz, POC*C*) ppm; ^31^P NMR (243 MHz, CD_3_OD): *δ* = 29.30 ppm.

##### *Diethyl 3*-*[2*-*(5*-*fluoro*-*3,4*-*dihydro*-*2,4*-*dioxopyrimidin*-*1(2H)*-*yl)acetamido]propylphosphonate* (**18c**, C_13_H_21_FN_3_O_6_P × 0.5H_2_O)

The crude product obtained from 0.055 g diethyl 3-aminopropylphosphonate (**7c**, 0.28 mmol) and 0.053 g (5-fluorouracil-1-yl)acetic acid (**10**, 0.28 mmol) in the presence of 0.054 g EDC × HCl (0.28 mmol) and 0.040 cm^3^ TEA (0.28 mmol) according to the general procedure B was chromatographed with chloroform–methanol mixtures (50:1, 20:1 v/v) and the selected fractions were crystallized from a methanol–diethyl ether mixture to give pure compound **18c** (0.048 g, 47% yield) as a white solid. M.p.: 143–144 °C; IR (KBr): $\bar{\nu }$ = 3240, 3096, 3032, 2996, 2955, 2940, 2884, 2835, 1740, 1669, 1677, 1240, 1033 cm^−1^; ^1^H NMR (600 MHz, CD_3_OD): *δ* = 7.81 (d, *J* = 6.3 Hz, 1H, HC6), 4.40 (s, 2H, C(O)CH_2_), 4.17–4.07 (m, 4H, 2 × POC*H*_2_CH_3_), 3.33 (t, *J* = 6.4 Hz, 2H, PCH_2_CH_2_C*H*_2_), 1.88–1.77 (m, 4H, PC*H*_2_C*H*_2_), 1.35 (t, *J* = 7.0 Hz, 6H, 2 × POCH_2_C*H*_3_) ppm; ^13^C NMR (151 MHz, CD_3_OD): *δ* = 167.91, 158.58 (d, *J* = 25.7 Hz), 150.18, 140.20 (d, *J* = 232.5 Hz), 130.40 (d, *J* = 33.2 Hz), 61.90 (d, *J* = 6.6 Hz, POC), 50.01, 39.36 (d, *J* = 18.7 Hz, PCC*C*), 22.11 (d, *J* = 3.9 Hz, PC*C*), 21.91 (d, *J* = 141.9 Hz, PC), 15.32 (d, *J* = 5.7 Hz, POC*C*) ppm; ^31^P NMR (243 MHz, CD_3_OD): *δ* = 32.80 ppm.

##### *Diethyl 2*-*[2*-*(5*-*fluoro*-*3,4*-*dihydro*-*2,4*-*dioxopyrimidin*-*1(2H)*-*yl)acetamido)ethoxy]methylphosphonate* (**18d**, C_13_H_21_FN_3_O_7_P)

The crude product obtained from 0.076 g diethyl 3-aminopropylphosphonate (**18d**, 0.360 mmol) and 0.068 g (5-fluorouracil-1-yl)acetic acid (**10**, 0.36 mmol) in the presence of 0.069 g EDC × HCl (0.36 mmol) and 0.050 cm^3^ TEA (0.360 mmol) according to the general procedure B was chromatographed with chloroform–methanol mixtures (50:1, 20:1 v/v) and the selected fractions were crystallized from ethyl acetate to give pure compound **18d** (0.83 g, 61% yield) as a white solid. M.p.: 69–70 °C; IR (KBr): $\bar{\nu }$ = 3362, 3169, 3058, 2995, 2933, 2825, 1699, 1662, 1242, 1024, 974 cm^−1^; ^1^H NMR (200 MHz, CD_3_OD): *δ* = 7.83 (d, *J* = 6.2 Hz, 1H, HC6), 4.44 (s, 2H, C(O)CH_2_), 4.29–4.14 (m, 4H, 2 × POC*H*_2_CH_3_), 3.92 (d, *J* = 8.5 Hz, 2H, PCH_2_O), 3.70 (t, *J* = 5.3 Hz, 2H, PCH_2_OC*H*_2_CH_2_), 3.46 (t, *J* = 5.3 Hz, 2H, PCOCH_2_C*H*_2_), 1.38 (t, *J* = 7.1 Hz, 6H, 2 × POCH_2_C*H*_3_) ppm; ^13^C NMR (151 MHz, CD_3_OD): *δ* = 167.89, 158.09 (d, *J* = 25.7 Hz), 150.18, 140.19 (d, *J* = 232.5 Hz), 130.36 (d, *J* = 33.2 Hz), 71.43 (d, *J* = 12.1 Hz, PCO*C*), 64.18 (d, *J* = 167.6 Hz, PC), 62.79 (d, *J* = 6.6 Hz, PO*C*), 49.70, 38.97, 15.36 (d, *J* = 5.6 Hz, POC*C*) ppm; ^31^P NMR (81 MHz, CD_3_OD): *δ* = 23.11 ppm.

##### *Diethyl [2*-*(5*-*bromo*-*3,4*-*dihydro*-*2,4*-*dioxopyrimidin*-*1(2H)*-*yl)acetamido]methylphosphonate* (**19a**, C_11_H_17_BrN_3_O_6_P)

The crude product obtained from 0.099 g diethyl aminomethylphosphonate (**7a**, 0.59 mmol) and 0.088 g, (5-bromouracil-1-yl)acetic acid (**11**, 0.59 mmol) in the presence of 0.114 g EDC × HCl (0.592 mmol) and 0.083 cm^3^ TEA (0.59 mmol) according to the general procedure B was chromatographed with chloroform–methanol mixtures (50:1, 20:1 v/v) and the selected fractions were crystallized from an ethanol–diethyl ether mixture to give pure compound **19a** (0.094 g, 40% yield) as a white solid. M.p.: 211–213 °C; IR (KBr): $\bar{\nu }$ = 3225, 3065, 2992, 2941, 2830, 1698, 1680, 1024, 623 cm^−1^; ^1^H NMR (200 MHz, CD_3_OD): *δ* = 8.04 (s, 1H, HC6), 4.53 (d, *J* = 1.5 Hz, 2H, C(O)CH_2_), 4.26–4.11 (m, 4H, 2 × POC*H*_2_CH_3_), 3.78 (d, *J* = 11.8 Hz, 2H, PCH_2_), 1.37 (t, *J* = 7.1 Hz, 6H, 2 × POCH_2_C*H*_3_) ppm; ^13^C NMR (151 MHz, CD_3_OD): *δ* = 167.70 (d, *J* = 3.6 Hz), 160.72, 150.80, 145.79, 95.09, 62.83 (d, *J* = 6.0 Hz, POC), 49.60, 34.27 (d, *J* = 158.6 Hz, PC), 15.29 (d, *J* = 5.7 Hz, POC*C*) ppm; ^31^P NMR (273 MHz, CD_3_OD): *δ* = 22.70 ppm.

##### *Diethyl 2*-*[2*-*(5*-*bromo*-*3,4*-*dihydro*-*2,4*-*dioxopyrimidin*-*1(2H)*-*yl)acetamido]ethylphosphonate* (**19b**, C_12_H_19_BrN_3_O_6_P)

The crude product obtained from 0.078 g diethyl 2-aminoethylphosphonate (**7b**, 0.431 mmol) and 0.107 g (5-bromouracil-1-yl)acetic acid (**11**, 0.431 mmol) in the presence of 0.083 g EDC × HCl (0.431 mmol) and 0.060 cm^3^ TEA (0.431 mmol) according to the general procedure B was chromatographed with chloroform–methanol mixtures (100:1, 50:1, 20:1 v/v) and the selected fractions were crystallized from ethyl acetate to give pure compound **19b** (0.082 g, 46% yield) as white needles. M.p.: 211–213 °C; IR (KBr): $\bar{\nu }$ = 3224, 3164, 3067, 2994, 2822, 1708, 1680, 1216, 1019, 619 cm^−1^; ^1^H NMR (200 MHz, CD_3_OD): *δ* = 8.04 (s, 1H, HC6), 4.46 (s, 2H, C(O)CH_2_), 4.24–4.07 (m, 4H, 2 × POC*H*_2_CH_3_), 3.56-3.43 (m, 2H, PCH_2_C*H*_2_), 2.20–2.04 (m, 2H, 2H, PC*H*_2_), 1.38 (t, *J* = 7.1 Hz, 3H, POCH_2_C*H*_3_) ppm; ^13^C NMR (151 MHz, CD_3_OD): *δ* = 167.68, 160.72, 150.89, 145.78, 95.17, 62.09 (d, *J* = 6.6 Hz, POC), 49.83, 33.43 (d, *J* = 1.7 Hz), 24.86 (d, *J* = 139.5 Hz, PC), 15.29 (d, *J* = 5.7 Hz, POC*C*) ppm; ^31^P NMR (81 MHz, CD_3_OD): *δ* = 30.20 ppm.

##### *Diethyl 3*-*[2*-*(5*-*bromo*-*3,4*-*dihydro*-*2,4*-*dioxopyrimidin*-*1(2H)*-*yl)acetamido]propylphosphonate* (**19c**, C_13_H_21_BrN_3_O_6_P)

The crude product obtained from 0.151 g diethyl 3-aminopropylphosphonate (**7c**, 0.774 mmol) and 0.115 g (5-bromouracil-1-yl)acetic acid (**11**, 0.774 mmol) in the presence of 0.148 g EDC × HCl (0.774 mmol) and 0.108 cm^3^ TEA (0.774 mmol) according to the general procedure B was chromatographed with dichloromethane–methanol mixtures (100:1, 50:1, 20:1 v/v) and the selected fractions were crystallized from a methanol–diethyl ether mixture to give pure compound **19c** (0.063 g, 20% yield) as a white solid. M.p.: 164–166 °C; IR (KBr): $\bar{\nu }$ = 3259, 3039, 2830, 2835, 1720, 1681, 1235, 1033 cm^−1^; ^1^H NMR (600 MHz, CD_3_OD): *δ* = 8.05 (s, 1H, HC6), 4.46 (s, 2H, C(O)CH_2_), 4.21–4.04 (m, 4H, 2 × POC*H*_2_CH_3_), 3.33 (t, *J* = 6.4 Hz, 2H, PCH_2_CH_2_C*H*_2_), 1.98–1.72 (m, 4H, PC*H*_2_C*H*_2_), 1.36 (t, *J* = 7.0 Hz, 6H, 2 × POCH_2_C*H*_3_) ppm; ^13^C NMR (151 MHz, CD_3_OD): *δ* = 167.84, 160.77, 150.87, 145.88, 95.09, 61.89 (d, *J* = 6.6 Hz, POC), 50.05, 39.36 (d, *J* = 18.7 Hz, PCC*C*), 22.09 (d, *J* = 4.7 Hz, PC*C*), 21.90 (d, *J* = 141.9 Hz, PC), 15.30 (d, *J* = 7.6 Hz, POC*C*) ppm; ^31^P NMR (81 MHz, CD_3_OD): *δ* = 33.70 ppm.

##### *Diethyl 2*-*[2*-*(5*-*bromo*-*3,4*-*dihydro*-*2,4*-*dioxopyrimidin*-*1(2H)*-*yl)acetamido)ethoxy]methylphosphonate* (**19d**, C_13_H_21_BrN_3_O_7_P)

The crude product obtained from 0.170 g diethyl (2-aminoethoxy)methylphosphonate (**7d**, 0.805 mmol) and 0.200 g (5-bromouracil-1-yl)acetic acid (**11**, 0.805 mmol) in the presence of 0.154 g EDC × HCl (0.805 mmol) and 0.112 cm^3^ TEA (0.805 mmol) according to the general procedure C for 1.0 h was chromatographed with chloroform–methanol mixtures (50:1, 20:1 v/v) and the selected fractions were crystallized from ethyl acetate to give pure compound **19d** (0.172 g, 48% yield) as a white powder. M.p.: 149–151 °C; IR (KBr): $\bar{\nu }$ = 3323, 3107, 2990, 2867, 2820, 2768, 1711, 1669, 1237, 1034, 972 cm^−1^; ^1^H NMR (200 MHz, CD_3_OD): *δ* = 8.04 (s, 1H, HC6), 4.49 (s, 2H, C(O)CH_2_), 4.29–4.14 (m, 4H, 2 × POC*H*_2_CH_3_), 3.92 (d, *J* = 8.5 Hz, 2H, PCH_2_O), 3.69 (t, *J* = 5.3 Hz, 2H, PCH_2_OC*H*_2_CH_2_), 3.46 (t, *J* = 5.3 Hz, 2H, PCOCH_2_C*H*_2_), 1.38 (t, *J* = 7.1 Hz, 6H, 2 × POCH_2_C*H*_3_) ppm; ^13^C NMR (151 MHz, CD_3_OD): *δ* = 167.82, 160.77, 150.88, 145.90, 95.06, 71.44 (d, *J* = 12.0 Hz, PCO*C*), 64.19 (d, *J* = 167.6 Hz, PC), 62.80 (d, *J* = 6.6 Hz, PO*C*), 49.77, 39.00, 15.40 (d, *J* = 5.6 Hz, POC*C*) ppm; ^31^P NMR (81 MHz, CD_3_OD): *δ* = 23.11 ppm.

##### *Diethyl [2*-*(3,4*-*dihydro*-*5*-*methyl*-*2,4*-*dioxopyrimidin*-*1(2H)*-*yl)acetamido]methylphosphonate* (**20a**, C_13_H_22_N_3_O_6_P)

The crude product obtained from 0.048 g diethyl aminomethylphosphonate (**7a**, 0.287 mmol) and 0.053 g (thymine-1-yl)acetic acid (**12**, 0.287 mmol) in the presence of 0.055 g EDC × HCl (0.287 mmol) and 0.040 cm^3^ TEA (0.287 mmol) according to the general procedure C was chromatographed with chloroform–methanol mixtures (50:1, 20:1 v/v) to give pure compound **20a** (0.060 g, 63% yield) as a white powder. M.p.: 211.5–212.5 °C; IR (KBr): $\bar{\nu }$ = 3227, 3058, 2986, 2932, 2833, 1697, 1653, 1242, 1200, 1020 cm^−1^; ^1^H NMR (200 MHz, CD_3_OD): *δ* = 7.41 (q, ^4^ *J* = 1.2 Hz, 1H, HC6), 4.48 (d, *J* = 1.5 Hz, 2H, C(O)CH_2_), 4.28–4.11 (m, 4H, 2 × POC*H*_2_CH_3_), 3.77 (d, *J* = 11.8 Hz, 2H, PCH_2_), 1.91 (d, ^4^ *J* = 1.2 Hz, 3H, CH_3_), 1.37 (t, *J* = 6.8 Hz, 6H, 2 × POCH_2_C*H*_3_) ppm; ^13^C NMR (151 MHz, CD_3_OD): *δ* = 168.11 (d, ^3^ *J* = 4.2 Hz, *C*(O)NHCP), 165.58, 151.57, 142.15, 109.66, 62.80 (d, *J* = 6.5 Hz, POC), 49.39, 34.23 (d, *J* = 158.5 Hz, PC), 15.28 (d, *J* = 5.7 Hz, POC*C*), 10.78 ppm; ^31^P NMR (81 MHz, CD_3_OD): *δ* = 23.6 ppm.

##### *Diethyl 2*-*[2*-*(3,4*-*dihydro*-*5*-*methyl*-*2,4*-*dioxopyrimidin*-*1(2H)*-*yl)acetamido]ethylphosphonate* (**20b**, C_13_H_22_N_3_O_6_P)

The crude product obtained from 0.100 g diethyl 2-aminoethylphosphonate (**7b**, 0.550 mmol) and 0.101 g (thymine-1-yl)acetic acid (**12**, 0.550 mmol) in the presence of 0.105 g EDC × HCl (0.550 mmol) and 0.077 cm^3^ TEA (0.55 mmol) according to the general procedure B was chromatographed with chloroform–methanol mixtures (100:1, 50:1, 20:1 v/v) to give pure compound **20b** (0.141 g, 74% yield) as a white powder. M.p.: 196–197 °C; IR (KBr): $\bar{\nu }$ = 3229, 3161, 3035, 2979, 2930, 2827, 1695, 1651, 1230, 1022 cm^−1^; ^1^H NMR (600 MHz, CD_3_OD): *δ* = 7.35 (s, 1H, HC6), 4.37 (s, 2H, C(O)CH_2_), 4.16–4.07 (m, 4H, 2 × POC*H*_2_CH_3_), 3.45 (dt, *J* = 12.2 Hz, *J* = 7.6 Hz, 2H, PCH_2_C*H*_2_), 2.07 (dt, *J* = 18.2 Hz, *J* = 7.6 Hz, 2H, PC*H*_2_), 1.88 (s, 3H, CH_3_), 1.34 (t, *J* = 7.1 Hz, 6H, 2 × POCH_2_C*H*_3_) ppm; ^13^C NMR (151 MHz, CD_3_OD): *δ* = 168.10, 165.57, 151.65, 142.24, 109.71, 62.10 (d, *J* = 6.5 Hz, POC), 49.68, 33.39 (d, *J* = 1.5 Hz), 24.88 (d, *J* = 138.9 Hz, PC), 15.34 (d, *J* = 6.0 Hz, POC*C*), 10.82 ppm; ^31^P NMR (243 MHz, CD_3_OD): *δ* = 33.29 ppm.

##### *Diethyl 3*-*[2*-*(3,4*-*dihydro*-*5*-*methyl*-*2,4*-*dioxopyrimidin*-*1(2H)*-*yl)acetamido]propylphosphonate* (**20c**, C_14_H_24_N_3_O_6_P × 0.5H_2_O)

The crude product obtained from 0.050 g diethyl 3-aminopropylphosphonate (**7c**, 0.26 mmol) and 0.047 g (thymine-1-yl)acetic acid (**12**, 0.26 mmol) in the presence of 0.049 g EDC × HCl (0.26 mmol) and 0.036 cm^3^ TEA (0.26 mmol) according to the general procedure B was chromatographed with chloroform–methanol mixtures (50:1, 20:1 v/v) and the selected fractions were crystallized from a methanol–diethyl ether mixture to give pure compound **20c** (0.043 g, 46% yield) as a white solid. M.p.: 183–184 °C; IR (KBr): $\bar{\nu }$ = 3264, 3158, 3093, 3035, 2982, 2944, 2884, 2830, 1698, 1683, 1238, 1016 cm^−1^; ^1^H NMR (600 MHz, CD_3_OD): *δ* = 7.38 (q, *J* = 1.3 Hz, 1H, HC6), 4.40 (s, 2H, C(O)CH_2_), 4.16–4.07 (m, 4H, 2 × POC*H*_2_CH_3_), 3.30 (t, *J* = 6.9 Hz, 2H, PCH_2_CH_2_C*H*_2_), 1.90 (d, *J* = 1.3 Hz, 3H, CH_3_), 1.88–1.77 (m, 4H, PC*H*_2_C*H*_2_), 1.35 (t, *J* = 7.1 Hz, 6H, 2 × POCH_2_C*H*_3_) ppm; ^13^C NMR (151 MHz, CD_3_OD): *δ* = 168.26, 165.61, 151.62, 142.25, 109.65, 61.87 (d, *J* = 6.6 Hz, POC), 49.84, 39.34 (d, *J* = 18.1 Hz, PCC*C*), 22.13 (d, *J* = 4.7 Hz, PC*C*), 21.92 (d, *J* = 141.9 Hz, PC), 15.30 (d, *J* = 6.2 Hz, POC*C*), 10.78 ppm; ^31^P NMR (243 MHz, CD_3_OD): *δ* = 32.78 ppm.

##### *Diethyl 2*-*[2*-*(3,4*-*dihydro*-*5*-*methyl*-*2,4*-*dioxopyrimidin*-*1(2H)*-*yl)acetamido)ethoxy]methylphosphonate* (**20d**, C_14_H_24_N_3_O_7_P)

The crude product obtained from 0.072 g diethyl (2-aminoethoxy)methylphosphonate (**7d**, 0.34 mmol) and 0.063 g (thymine-1-yl)acetic acid (**12**, 0.34 mmol) in the presence of 0.065 g EDC × HCl (0.34 mmol) and 0.048 cm^3^ TEA (0.34 mmol) according to the general procedure B was chromatographed with chloroform–methanol mixtures (50:1, 20:1 v/v) and the selected fractions were crystallized from ethyl acetate to give pure compound **20d** (0.060 g, 47% yield) as a white solid. M.p.: 116–117 °C; IR (KBr): $\bar{\nu }$ = 3323, 3151, 3078, 3057, 2982, 2954, 2906, 2837, 1678, 1661, 1236, 1026 cm^−1^; ^1^H NMR (200 MHz, CD_3_OD): *δ* = 7.40 (q, *J* = 1.1 Hz, 1H, HC6), 4.44 (s, 2H, C(O)CH_2_), 4.29–4.14 (m, 4H, 2 × POC*H*_2_CH_3_), 3.93 (d, *J* = 8.5 Hz, 2H, PCH_2_O), 3.69 (t, *J* = 5.3 Hz, 2H, PCH_2_OC*H*_2_CH_2_), 3.46 (t, *J* = 5.3 Hz, 2H, PCOCH_2_C*H*_2_), 1.92 (d, *J* = 1.1 Hz, 3H, CH_3_), 1.38 (t, *J* = 7.0 Hz, 6H, 2 × POCH_2_C*H*_3_) ppm; ^13^C NMR (151 MHz, CD_3_OD): *δ* = 168.23, 165.60, 151.62, 142.26, 109.62, 71.45 (d, *J* = 12.1 Hz, PCO*C*), 64.17 (d, *J* = 167.6 Hz, PC), 62.78 (d, *J* = 6.7 Hz, PO*C*), 49.54, 38.98, 15.37 (d, *J* = 5.6 Hz, POC*C*), 10.79 ppm; ^31^P NMR (81 MHz, CD_3_OD): *δ* = 23.10 ppm.

##### *Diethyl [2*-*[4*-*[[(benzyloxy)carbonyl]amino]*-*2*-*oxopyrimidin*-*1(2H)*-*yl]acetamido]methylphosphonate* (**21a**, C_19_H_25_N_4_O_7_P × 0.5H_2_O)

The crude product obtained from 0.104 g diethyl aminomethylphosphonate (**7a**, 0.622 mmol) and 0.189 g [*N* ^4^-[(benzyloxy)carbonyl]cytosine-1-yl]acetic acid (**13**, 0.622 mmol) in the presence of 0.119 g EDC × HCl (0.622 mmol) and 0.087 cm^3^ TEA (0.62 mmol) according to the general procedure B was chromatographed with chloroform–methanol mixtures (100:1, 50:1, 20:1 v/v) and the selected fractions were crystallized from a methanol–petroleum ether mixture to give pure compound **21a** (0.125 g, 44% yield) as a white solid. M.p.: 180–182 °C; IR (KBr): $\bar{\nu }$ = 3279, 3147, 3081, 2990, 2949, 2928, 1753, 1657, 1257, 1205, 1025 cm^−1^; ^1^H NMR (600 MHz, CDCl_3_): *δ* = 9.18 (brs, 1H, HNCbz), 8.29 (brt, *J* = 5.9 Hz, 1H, HNC(O)), 7.66 (d, *J* = 7.1 Hz, 1H, HC6), 7.41–7.29 (m, 5H), 7.25 (brs, 1H), 5.22 (s, 2H, C*H*_2_C_6_H_5_), 4.65 (s, 2H, C(O)CH_2_), 4.12–4.07 (m, 4H, 2 × POC*H*_2_CH_3_), 3.75 (dd, *J* = 11.8 Hz, *J* = 5.9 Hz, 2H, PCH_2_), 1.28 (t, *J* = 7.0 Hz, 6H, 2 × POCH_2_C*H*_3_) ppm; ^13^C NMR (151 MHz, CDCl_3_): *δ* = 166.89 (d, *J* = 4.6 Hz), 163.24, 156.18, 152.72, 149.37, 135.32, 128.61, 128.50, 128.19, 95.55, 67.70, 62.78 (d, *J* = 6.6 Hz, POC), 52.31, 35.07 (d, *J* = 157.5 Hz, PC), 16.34 (d, *J* = 5.6 Hz, POC*C*) ppm; ^31^P NMR (273 MHz, CDCl_3_): *δ* = 22.49 ppm.

##### *Diethyl 2*-*[2*-*[4*-*[[(benzyloxy)carbonyl]amino]*-*2*-*oxopyrimidin*-*1(2H)*-*yl]acetamido]ethylphosphonate* (**21b**, C_20_H_27_N_4_O_7_P)

The crude product obtained from 0.111 g diethyl 2-aminoethylphosphonate (**7b**, 0.613 mmol) and 0.186 g [*N* ^4^-[(benzyloxy)carbonyl]cytosine-1-yl]acetic acid (**13**, 0.613 mmol) in the presence of 0.117 g EDC × HCl (0.613 mmol) and 0.085 cm^3^ TEA (0.61 mmol) according to the general procedure B was chromatographed with chloroform–methanol mixtures (100:1, 50:1, 20:1 v/v) to give pure compound **21b** (0.224 g, 78% yield) as a white solid. M.p.: 127–128 °C; IR (KBr): $\bar{\nu }$ = 3241, 3063, 3035, 2982, 2930, 1742, 1657, 1630, 1215, 1056 cm^−1^; ^1^H NMR (600 MHz, CD_3_OD): *δ* = 7.92 (d, *J* = 7.3 Hz, 1H, HC6), 7.44–7.33 (m, 5H), 7.30 (d, *J* = 7.3 Hz, 1H, HC5), 5.25 (s, 2H, C*H*_2_C_6_H_5_), 4.56 (s, 2H, C(O)CH_2_), 4.18–4.08 (m, 4H, 2 × POC*H*_2_CH_3_), 3.48 (dt, *J* = 11.7 Hz, *J* = 7.6 Hz, 2H, PCH_2_C*H*_2_), 2.11 (dt, *J* = 18.2 Hz, *J* = 7.6 Hz, 2H, PC*H*_2_), 1.35 (t, *J* = 7.1 Hz, 6H, 2 × POCH_2_C*H*_3_) ppm; ^13^C NMR (151 MHz, CD_3_OD): *δ* = 167.65, 164.11, 157.08, 153.17, 150.21, 135.80, 128.23, 128.06, 127.89, 95.39, 67.21, 62.08 (d, *J* = 6.5 Hz, POC), 51.96, 33.47, 24.89 (d, *J* = 139.5 Hz, PC), 15.34 (d, *J* = 5.6 Hz, POC*C*) ppm; ^31^P NMR (243 MHz, CD_3_OD): *δ* = 29.35 ppm.

##### *Diethyl 3*-*[2*-*[4*-*[[(benzyloxy)carbonyl]amino]*-*2*-*oxopyrimidin*-*1(2H)*-*yl]acetamido]propylphosphonate* (**21c**, C_21_H_29_N_4_O_7_P)

The crude product obtained from 0.050 g diethyl 3-aminopropylphosphonate (**7c**, 0.26 mmol) and 0.078 g [*N* ^4^-[(benzyloxy)carbonyl]cytosine-1-yl]acetic acid (**13**, 0.26 mmol) in the presence of 0.049 g EDC × HCl (0.26 mmol) and 0.036 cm^3^ TEA (0.26 mmol) according to the general procedure B was chromatographed with chloroform–methanol mixtures (50:1, 20:1 v/v) and the selected fractions were crystallized from an ethyl acetate–hexane mixture to give pure compound **21c** (0.066 g, 54% yield) as white needles. M.p.: 150–151 °C; IR (KBr): $\bar{\nu }$ = 3281, 3239, 3149, 3089, 3038, 2976, 2939, 1749, 1687, 1653, 1225, 1021 cm^−1^; ^1^H NMR (600 MHz, CD_3_OD): *δ* = 7.93 (d, *J* = 7.3 Hz, 1H, HC6), 7.45–7.35 (m, 5H), 7.31 (d, *J* = 7.3 Hz, 1H, HC5), 5.25 (s, 2H, C*H*_2_C_6_H_5_), 4.56 (s, 2H, C(O)CH_2_), 4.16–4.07 (m, 4H, 2 × POC*H*_2_CH_3_), 3.32 (t, *J* = 6.5 Hz, 2H, PCH_2_CH_2_C*H*_2_), 1.90–1.78 (m, 4H, PC*H*_2_C*H*_2_), 1.34 (t, *J* = 7.1 Hz, 6H, 2 × POCH_2_C*H*_3_) ppm; ^13^C NMR (151 MHz, CD_3_OD): *δ* = 167.79, 163.99, 157.04, 153.17, 150.28, 135.82, 128.23, 128.06, 127.89, 95.31, 67.20, 61.88 (d, *J* = 6.6 Hz, POC), 52.08, 39.43 (d, *J* = 18.7 Hz, PCC*C*), 22.12 (d, *J* = 4.9 Hz, PC*C*), 21.95 (d, *J* = 141.9 Hz, PC), 15.36 (d, *J* = 5.9 Hz, POC*C*) ppm; ^31^P NMR (243 MHz, CD_3_OD): *δ* = 32.77 ppm.

##### *Diethyl 2*-*[2*-*[4*-*[[(benzyloxy)carbonyl]amino]*-*2*-*oxopyrimidin*-*1(2H)*-*yl]acetamido] ethoxy]methylphosphonate* (**21d**, C_21_H_29_N_4_O_8_P)

The crude product obtained from 0.068 g diethyl (2-aminoethoxy)methylphosphonate (**7d**, 0.32 mmol) and 0.098 g [*N* ^4^-[(benzyloxy)carbonyl]cytosine-1-yl]acetic acid (**13**, 0.32 mmol) in the presence of 0.062 g EDC × HCl (0.32 mmol) and 0.045 cm^3^ TEA (0.32 mmol) according to the general procedure B was chromatographed with chloroform–methanol mixtures (50:1, 20:1 v/v) and the selected fractions were crystallized from an ethyl acetate–hexane mixture to give pure compound **21d** (0.073 g, 48% yield) as a white solid. M.p.: 129–131 °C; IR (KBr): $\bar{\nu }$ = 3332, 3145, 3069, 3032, 2981, 2930, 2869, 1751, 1658, 1214, 1022 cm^−1^; ^1^H NMR (200 MHz, CD_3_OD): *δ* = 7.96 (d, *J* = 7.4 Hz, 1H, HC6), 7.50–7.34 (m, 5H), 7.34 (d, *J* = 7.4 Hz, 1H, HC5), 5.27 (s, 2H, C*H*_2_C_6_H_5_), 4.61 (s, 2H, C(O)CH_2_), 4.28–4.14 (m, 4H, 2 × POC*H*_2_CH_3_), 3.93 (d, *J* = 8.5 Hz, 2H, PCH_2_O), 3.69 (t, *J* = 5.3 Hz, 2H, PCH_2_OC*H*_2_CH_2_), 3.46 (t, *J* = 5.3 Hz, 2H, PCOCH_2_C*H*_2_), 1.38 (t, *J* = 7.0 Hz, 6H, 2 × POCH_2_C*H*_3_) ppm; ^13^C NMR (151 MHz, CD_3_OD): *δ* = 167.77, 163.97, 157.06, 153.17, 150.26, 135.82, 128.23, 128.06, 127.89, 95.29, 71.48 (d, *J* = 12.0 Hz, PCO*C*), 67.21, 64.21 (d, *J* = 166.1 Hz, PC), 62.79 (d, *J* = 6.6 Hz, PO*C*), 51.82, 39.00, 15.41 (d, *J* = 5.8 Hz, POC*C*) ppm; ^31^P NMR (81 MHz, CD_3_OD): *δ* = 23.10 ppm.

##### *Diethyl [2*-*(3,4*-*dihydro*-*2,4*-*dioxoquinazolin*-*1(2H)*-*yl)acetamido]methylphosphonate* (**22a**, C_15_H_20_N_3_O_6_P × 2H_2_O)

The crude product obtained from 0.058 g diethyl aminomethylphosphonate (**7a**, 0.35 mmol) and 0.076 g 2-(3,4-dihydro-2,4-dioxoquinazolin-1-yl)acetic acid (**14**, 0.35 mmol) in the presence of 0.067 g EDC × HCl (0.35 mmol) and 0.048 cm^3^ TEA (0.35 mmol) according to the general procedure C was chromatographed with chloroform–methanol mixtures (50:1, 20:1 v/v) and the selected fractions were crystallized from a methanol–diethyl ether mixture to give pure compound **22a** (0.086 g, 67% yield) as a white powder. M.p.: 203–205 °C; IR (KBr): $\bar{\nu }$ = 3462, 3264, 3197, 2975, 2909, 1736, 1638, 1044, 1013 cm^−1^; ^1^H NMR (200 MHz, CD_3_OD): *δ* = 8.10–8.05 (m, 1H), 7.75–7.66 (m, 1H), 7.32-7.21 (m, 2H), 4.76 (d, *J* = 1.6 Hz, 2H, C(O)CH_2_), 4.27–4.12 (m, 4H, 2 × POC*H*_2_CH_3_), 3.78 (d, *J* = 11.8 Hz, 2H, PCH_2_), 1.37 (t, *J* = 6.8 Hz, 6H, 2 × POCH_2_C*H*_3_) ppm; ^13^C NMR (151 MHz, CD_3_OD): *δ* = 166.47 (d, *J* = 4.0 Hz, *C*(O)NHCP), 162.72, 150.78, 139.58, 135.05, 127.50, 122.72, 114.90, 113.99, 62.86 (d, *J* = 6.6 Hz, POC), 42.31, 34.23 (d, *J* = 158.4 Hz, PC), 15.30 (d, *J* = 5.6 Hz, POC*C*) ppm; ^31^P NMR (81 MHz, CD_3_OD): *δ* = 23.56 ppm.

##### *Diethyl 2*-*[2*-*(3,4*-*dihydro*-*2,4*-*dioxoquinazolin*-*1(2H)*-*yl)acetamido]ethylphosphonate* (**22b**, C_16_H_22_N_3_O_6_P)

The crude product obtained from 0.051 g diethyl 2-aminoethylphosphonate (**7b**, 0.28 mmol) and 0.062 g 2-(3,4-dihydro-2,4-dioxoquinazolin-1-yl)acetic acid (**14**, 0.28 mmol) in the presence of 0.054 g EDC × HCl (0.28 mmol) and 0.039 cm^3^ TEA (0.28 mmol) according to the general procedure C was chromatographed with chloroform–methanol mixtures (100:1, 50:1, 20:1 v/v) to give pure compound **22b** (0.073 g, 68% yield) as a white powder. M.p.: 180–182 °C; IR (KBr): $\bar{\nu }$ = 3249, 3205, 3083, 2983, 2930, 2882, 1736, 1637, 1027 cm^−1^; ^1^H NMR (600 MHz, CD_3_OD): *δ* = 8.64 (dd, *J* = 7.1 Hz, *J* = 1.3 Hz, 1H), 7.69 (dt, 1H, *J* = 7.1 Hz, *J* = 1.0 Hz), 7.27 (dt, *J* = 8.2 Hz, *J* = 1.0 Hz, 1H), 7.22 (d, *J* = 8.2 Hz, 1H), 4.69 (s, 2H, C(O)CH_2_), 4.19–4.09 (m, 4H, 2 × POC*H*_2_CH_3_), 3.50–3.46 (m, 2H, PCH_2_C*H*_2_), 2.14–2.08 (m, 2H, PC*H*_2_), 1.36 (t, *J* = 7.1 Hz, 6H, 2 × POCH_2_C*H*_3_) ppm; ^13^C NMR (151 MHz, CD_3_OD): *δ* = 168.55, 162.76, 150.80, 139.55, 135.04, 127.50, 122.73, 114.90, 113.99, 62.06 (d, *J* = 6.6 Hz, POC), 42.55 (PC*C*), 33.40, 26.64, 24.91 (d, *J* = 138.9 Hz, PC), 15.32 (d, *J* = 6.0 Hz, POC*C*) ppm; ^31^P NMR (243 MHz, CD_3_OD): *δ* = 29.51 ppm.

##### *Diethyl 3*-*[2*-*(3,4*-*dihydro*-*2,4*-*dioxoquinazolin*-*1(2H)*-*yl)acetamido]propylphosphonate* (**22c**, C_17_H_24_N_3_O_6_P)

The crude product obtained from 0.085 g diethyl 3-aminopropylphosphonate (**7c**, 0.435 mmol) and 0.096 g 2-(3,4-dihydro-2,4-dioxoquinazolin-1-yl)acetic acid (**14**, 0.435 mmol) in the presence of 0.083 g EDC × HCl (0.435 mmol) and 0.061 cm^3^ TEA (0.435 mmol) according to the general procedure B was chromatographed with chloroform–methanol mixtures (50:1, 20:1 v/v) to give pure compound **22c** (0.083 g, 48% yield) as a white powder. M.p.: 168–169 °C; IR (KBr): $\bar{\nu }$ = 3267, 3201, 3148, 3065, 2984, 2928, 1736, 1661, 1638, 1232, 1031 cm^−1^; ^1^H NMR (200 MHz, CD_3_OD): *δ* = 8.07–8.02 (m, 1H), 7.73–7.64 (m, 1H), 7.30–7.17 (m, 2H), 4.70 (s, 2H, C(O)CH_2_), 4.23–4.05 (m, 4H, 2 × POC*H*_2_CH_3_), 3.33 (t, *J* = 6.5 Hz, 2H, PCH_2_CH_2_C*H*_2_), 1.98–1.67 (m, 4H, PC*H*_2_C*H*_2_), 1.36 (t, *J* = 7.1 Hz, 6H, 2 × POCH_2_C*H*_3_) ppm; ^13^C NMR (151 MHz, CD_3_OD): *δ* = 168.74, 162.82, 150.84, 139.61, 135.01, 127.50, 122.68, 114.88, 114.07, 61.86 (d, *J* = 6.6 Hz, POC), 42.60, 39.24 (d, *J* = 19.0 Hz, PCC*C*), 22.17 (d, *J* = 4.5 Hz, PC*C*), 21.85 (d, *J* = 141.9 Hz, PC), 15.30 (d, *J* = 6.3 Hz, POC*C*) ppm; ^31^P NMR (81 MHz, CD_3_OD): *δ* = 33.86 ppm.

##### *Diethyl 2*-*[2*-*(3,4*-*dihydro*-*2,4*-*dioxoquinazolin*-*1(2H)*-*yl)acetamido]ethoxy]methylphosphonate* (**22d**, C_17_H_24_N_3_O_7_P)

The crude product obtained from 0.078 g diethyl (2-aminoethoxy)methylphosphonate (**7d**, 0.37 mmol) and 0.061 g 2-(3,4-dihydro-2,4-dioxoquinazolin-1-yl)acetic acid (**14**, 0.37 mmol) in the presence of 0.071 g EDC × HCl (0.37 mmol) and 0.051 cm^3^ TEA (0.37 mmol) according to the general procedure C was chromatographed with chloroform–methanol mixtures (50:1, 20:1 v/v) to give pure compound **22d** (0.127 g, 83% yield) as a white solid. M.p.: 169–170 °C; IR (KBr): $\bar{\nu }$ = 3196, 3148, 3123, 3065, 2978, 2940, 1737, 1639, 1240, 1028 cm^−1^; ^1^H NMR (200 MHz, CD_3_OD): *δ* = 8.09–8.04 (m, 1H), 7.74-7.66 (m, 1H), 7.32–7.21 (m, 2H), 4.72 (s, 2H, C(O)CH_2_), 4.29–4.14 (m, 4H, 2 × POC*H*_2_CH_3_), 3.94 (d, *J* = 8.5 Hz, 2H, PCH_2_O), 3.70 (t, *J* = 5.3 Hz, 2H, PCH_2_OC*H*_2_CH_2_), 3.46 (t, *J* = 5.3 Hz, 2H, PCOCH_2_C*H*_2_), 1.38 (t, *J* = 7.0 Hz, 3H, POCH_2_C*H*_3_) ppm; ^13^C NMR (151 MHz, CD_3_OD): *δ* = 168.64, 162.78, 150.82, 139.58, 135.00, 127.50, 122.69, 114.89, 114.04, 71.55 (d, *J* = 12.1 Hz, PCO*C*), 64.25 (d, *J* = 166.3 Hz, PC), 62.84 (d, *J* = 6.6 Hz, PO*C*), 42.47, 39.00, 15.39 (d, *J* = 5.6 Hz, POC*C*) ppm; ^31^P NMR (81 MHz, CD_3_OD): *δ* = 23.08 ppm.

##### *Diethyl [2*-*[6*-*(bis(tert*-*butoxycarbonyl))amino*-*9H*-*purin*-*9*-*yl]acetamido]methylphosphonate* (**23a**, C_22_H_35_N_6_O_8_P)

The crude product obtained from 0.109 g diethyl aminomethylphosphonate (**7a**, 0.622 mmol) and 0.256 g 2-[6-[bis(*tert*-butoxycarbonyl)amino]-9*H*-purin-9-yl]acetic acid (**15**, 0.622 mmol) in the presence of 0.125 g EDC × HCl (0.622 mmol) and 0.091 cm^3^ TEA (0.62 mmol) according to the general procedure B was chromatographed with chloroform–methanol mixtures (100:1, 50:1, 20:1 v/v) and the selected fractions were crystallized from a methanol–petroleum ether mixture to give pure compound **23a** (0.183 g, 52% yield) as a white solid. M.p.: 128–130 °C; IR (KBr): $\bar{\nu }$ = 3256, 3109, 2980, 2934, 1735, 1699, 1672, 1049 cm^−1^; ^1^H NMR (600 MHz, CD_3_OD): *δ* = 8.85 (s, 1H), 8.54 (s, 1H), 5.19 (s, 2H, C(O)CH_2_), 4.19–4.14 (m, 4H, 2 × POC*H*_2_CH_3_), 3.78 (d, *J* = 11.8 Hz, 2H, PCH_2_), 1.41 (s, 18H, 6 × CH_3_), 1.33 (t, *J* = 7.0 Hz, 6H, 2 × POCH_2_C*H*_3_) ppm; ^13^C NMR (151 MHz, CD_3_OD): *δ* = 166.75 (d, *J* = 4.4 Hz, *C*(O)NHCP), 153.84, 151.66, 150.14, 149.47, 147.72, 128.37, 83.91, 62.81 (d, *J* = 6.5 Hz, POC), 45.14, 34.41 (d, *J* = 158.6 Hz, PC), 26.65, 15.36 (d, *J* = 5.7 Hz, POC*C*) ppm; ^31^P NMR (243 MHz, CD_3_OD): *δ* = 22.68 ppm.

##### *Diethyl 2*-*[2*-*[6*-*(bis(tert*-*butoxycarbonyl))amino*-*9H*-*purin*-*9*-*yl]acetamido]ethylphosphonate* (**23b**, C_23_H_37_N_6_O_8_P × 0.5 H_2_O)

The crude product obtained from 0.108 g diethyl 2-aminoethylphosphonate (**7b**, 0.596 mmol) and 0.235 g 2-[6-[bis(*tert*-butoxycarbonyl)amino]-9*H*-purin-9-yl]acetic acid (**15**, 0.596 mmol) in the presence of 0.114 g EDC × HCl (0.596 mmol) and 0.083 cm^3^ TEA (0.60 mmol) according to the general procedure B was chromatographed with chloroform–methanol mixtures (100:1, 50:1, 20:1 v/v) to give pure compound **23b** (0.155 g, 47% yield) as a colorless oil. IR (film): $\bar{\nu }$ = 3356, 3284, 3085, 2983, 2936, 1788, 1760, 1697, 1251, 1027 cm^−1^; ^1^H NMR (200 MHz, CD_3_OD): *δ* = 8.87 (s, 1H), 8.56 (s, 1H), 5.14 (s, 2H, C(O)CH_2_), 4.25–4.07 (m, 4H, 2 × POC*H*_2_CH_3_), 3.60–3.48 (m, 2H, PCH_2_C*H*_2_), 2.22–2.06 (m, 2H, 2H, PC*H*_2_), 1.43 (s, 18H, 6 × CH_3_), 1.37 (t, *J* = 7.1 Hz, 6H, 2 × POCH_2_C*H*_3_) ppm; ^13^C NMR (151 MHz, CD_3_OD): *δ* = 166.73, 153.85, 151.65, 150.17, 149.46, 147.72, 128.37, 83.91, 62.08 (d, *J* = 6.6 Hz, POC), 45.26, 33.57, 26.64, 24.89 (d, *J* = 138.9 Hz, PC), 15.37 (d, *J* = 5.7 Hz, POC*C*) ppm; ^31^P NMR (81 MHz, CD_3_OD): *δ* = 30.17 ppm.

##### *Diethyl 3*-*[2*-*[6*-*(bis(tert*-*butoxycarbonyl))amino*-*9H*-*purin*-*9*-*yl]acetamido]propylphosphonate* (**23c**, C_24_H_39_N_6_O_8_P × 0.5H_2_O)

The crude product obtained from 0.059 g diethyl 3-aminopropylphosphonate (**15**, 0.30 mmol) and 0.119 g 2-[6-[bis(*tert*-butoxycarbonyl)amino]-9*H*-purin-9-yl]acetic acid (**15**, 0.302 mmol) in the presence of 0.058 g EDC × HCl (0.30 mmol) and 0.042 cm^3^ TEA (0.30 mmol) according to the general procedure B was chromatographed with chloroform–methanol mixtures (100:1 v/v) to give pure compound **23c** (0.072 g, 42% yield) as an yellowish oil. IR (film): $\bar{\nu }$ = 3363, 3281, 3087, 2983, 2937, 1788, 1693, 1220, 1024 cm^−1^; ^1^H NMR (600 MHz, CD_3_OD): *δ* = 8.85 (s, 1H), 8.53 (s, 1H), 5.12 (s, 2H, C(O)CH_2_), 4.17–4.07 (m, 4H, 2 × POC*H*_2_CH_3_), 3.36 (t, *J* = 6.5 Hz, 2H, PCH_2_CH_2_C*H*_2_), 1.92–1.78 (m, 4H, PC*H*_2_C*H*_2_), 1.42 (s, 18H, 6 × CH_3_), 1.34 (t, *J* = 7.0 Hz, 6H, 2 × POCH_2_C*H*_3_) ppm; ^13^C NMR (151 MHz, CD_3_OD): *δ* = 166.90, 153.82, 151.65, 150.16, 149.46, 147.73, 128.43, 83.92, 61.88 (d, *J* = 6.6 Hz, POC), 45.42, 39.50 (d, *J* = 19.6 Hz, PCC*C*), 26.61, 22.14 (d, *J* = 4.6 Hz, PC*C*), 22.00 (d, *J* = 142.6 Hz, PC), 15.33 (d, *J* = 5.6 Hz, POC*C*) ppm; ^31^P NMR (243 MHz, CD_3_OD): *δ* = 32.68 ppm.

##### *Diethyl 2*-*[2*-*[6*-*(bis(tert*-*butoxycarbonyl))amino*-*9H*-*purin*-*9*-*yl]acetamido]ethoxy)methyl)phosphonate* (**23d**, C_24_H_39_N_6_O_9_P × 0.5H_2_O)

The crude product obtained from 0.070 g diethyl (2-aminoethoxy)methylphosphonate (**7d**, 0.33 mmol) and 0.130 g 2-[6-[bis(*tert*-butoxycarbonyl)amino]-9*H*-purin-9-yl]acetic acid (**15**, 0.33 mmol) in the presence of 0.064 g EDC × HCl (0.33 mmol) and 0.046 cm^3^ TEA (0.33 mmol) according to the general procedure B was chromatographed with chloroform–methanol mixtures (100:1, 50:1 v/v) to give pure compound **23d** (0.102 g, 52% yield) as a colorless oil. IR (film): $\bar{\nu }$ = 3282, 3086, 2982, 2935, 1786, 1757, 1220, 1028 cm^−1^; ^1^H NMR (200 MHz, CDCl_3_): *δ* = 8.83 (s, 1H), 8.26 (s, 1H), 7.69 (t, *J* = 6.0 Hz, 1H, C(O)N*H*CH_2_), 4.96 (s, 2H, C(O)CH_2_), 4.29–4.10 (m, 4H, 2 × POC*H*_2_CH_3_), 3.82 (d, *J* = 7.9 Hz, 2H, PCH_2_O), 3.70 (t, *J* = 4.6 Hz, 2H, PCH_2_OC*H*_2_CH_2_), 3.50 (t, *J* = 4.6 Hz, 2H, PCOCH_2_C*H*_2_), 1.43 (s, 18 H, 6 × CH_3_), 1.35 (t, *J* = 7.1 Hz, 6H, 2 × POCH_2_C*H*_3_) ppm; ^13^C NMR (151 MHz, CDCl_3_): *δ* = 165.64, 153.53, 152.02, 150.43, 150.34, 145.63, 128.44, 83.72, 72.22 (d, *J* = 8.8 Hz, PCO*C*), 65.59 (d, *J* = 166.8 Hz, PC), 62.69 (d, *J* = 6.7 Hz, PO*C*), 46.07, 39.76, 27.79, 16.46 (d, *J* = 5.5 Hz, POC*C*) ppm; ^31^P NMR (81 MHz, CDCl_3_): *δ* = 22.77 ppm.

##### *Diethyl [2*-*(2*-*amino*-*6*-*chloro*-*9H*-*purin*-*9*-*yl)acetamido]methylphosphonate* (**24a**, C_12_H_18_ClN_6_O_4_P × 0.5H_2_O)

The crude product obtained from 0.419 g diethyl aminomethylphosphonate (**7a**, 2.51 mmol) and 0.571 g 2-(2-amino-6-chloropurin-9-yl)acetic acid (**16**, 2.51 mmol) in the presence of 0.481 g EDC × HCl (2.51 mmol) and 0.358 cm^3^ TEA (2.51 mmol) according to the general procedure C was chromatographed with chloroform–methanol mixtures (50:1, 20:1 v/v) and the selected fractions were crystallized from a methanol–diethyl ether mixture to give pure compound **24a** (0.211 g, 22% yield) as a white solid. M.p.: 130–132 °C; IR (KBr): $\bar{\nu }$ = 3397, 3340, 3221, 3111, 3065, 2987, 2946, 1684, 1610, 1562, 1219, 1053, 1013 cm^−1^; ^1^H NMR (200 MHz, CD_3_OD): *δ* = 8.11 (s, 1H), 4.95 (d, *J* = 1.4 Hz, 2H, C(O)CH_2_), 4.21–4.10 (m, 4H, 2 × POC*H*_2_CH_3_), 3.78 (d, *J* = 11.8 Hz, 2H, PCH_2_), 1.34 (t, *J* = 7.0 Hz, 3H, POCH_2_C*H*_3_) ppm; ^13^C NMR (151 MHz, CD_3_OD): *δ* = 167.18 (d, *J* = 3.5 Hz), 160.32, 154.18, 150.17, 143.84, 123.22, 62.80 (d, *J* = 6.6 Hz, POC), 44.82, 34.37 (d, *J* = 158.6 Hz, PC), 15.26 (d, *J* = 6.0 Hz, POC*C*) ppm; ^31^P NMR (81 MHz, CD_3_OD): *δ* = 23.56 ppm.

##### *Diethyl 2*-*[2*-*(2*-*amino*-*6*-*chloro*-*9H*-*purin*-*9*-*yl)acetamido]ethylphosphonate* (**24b**, C_13_H_20_ClN_6_O_4_P)

The crude product obtained from 0.307 g diethyl 2-aminoethylphosphonate (**7b**, 1.69 mmol) and 0.386 g 2-(2-amino-6-chloropurin-9-yl)acetic acid (**16**, 1.69 mmol) in the presence of 0.325 g EDC × HCl (1.69 mmol) and 0.236 cm^3^ TEA (1.69 mmol) according to the general procedure C was chromatographed with chloroform–methanol mixtures (50:1, 30:1, 10:1 v/v) and the selected fractions were crystallized from a methanol–diethyl ether mixture to give pure compound **24b** (0.126 g, 19% yield) as a white solid. M.p.: 158–160 °C; IR (KBr): $\bar{\nu }$ = 3411, 3322, 3275, 3122, 3074, 2985, 2929, 1688, 1254, 1030 cm^−1^; ^1^H NMR (600 MHz, CD_3_OD): *δ* = 8.05 (s, 1H), 4.84 (s, 2H, C(O)CH_2_), 4.13–4.09 (m, 4H, 2 × POC*H*_2_CH_3_), 3.48-3.44 (m, 2H, PCH_2_C*H*_2_), 2.10–2.06 (m, 2H, 2H, PC*H*_2_), 1.32 (t, *J* = 7.0 Hz, 6H, 2 × POCH_2_C*H*_3_) ppm; ^13^C NMR (151 MHz, CD_3_OD): *δ* = 171.14, 164.26, 158.13, 154.12, 147.78, 127.21, 66.02 (d, *J* = 6.0 Hz, POC), 48.96, 37.43 (d, *J* = 1.7 Hz), 28.80 (d, *J* = 138.9 Hz, PC), 19.24 (d, *J* = 6.1 Hz, POC*C*) ppm; ^31^P NMR (243 MHz, CD_3_OD): *δ* = 33.23 ppm.

##### *Diethyl 3*-*[2*-*(2*-*amino*-*6*-*chloro*-*9H*-*purin*-*9*-*yl)acetamido]propylphosphonate* (**24c**, C_24_H_39_N_6_O_8_P)

The crude product obtained from 0.203 g diethyl 3-aminopropylphosphonate (**7c**, 1.04 mmol) and 0.237 g 2-(2-amino-6-chloropurin-9-yl)acetic acid (**16**, 1.04 mmol) in the presence of 0.199 g EDC × HCl (1.04 mmol) and 0.145 cm^3^ TEA (1.04 mmol) according to the general procedure C was chromatographed with dichloromethane–methanol mixtures (50:1, 20:1, 10:1 v/v) and the selected fractions were crystallized from an ethanol–ethyl acetate mixture to give pure compound **24c** (0.106 g, 25% yield) as a white solid. M.p.: 180–181 °C; IR (KBr): $\bar{\nu }$ = 3408, 3343, 3273, 3090, 2988, 2945, 2908, 2869, 1680, 1644, 1608, 1215, 1040 cm^−1^; ^1^H NMR (600 MHz, CD_3_OD): *δ* = 8.07 (s, 1H), 4.87 (s, 2H, C(O)CH_2_), 4.15–4.06 (m, 4H, 2 × POC*H*_2_CH_3_), 3.33 (t, *J* = 6.5 Hz, 2H, PCH_2_CH_2_C*H*_2_), 1.88–1.77 (m, 4H, PC*H*_2_C*H*_2_), 1.33 (t, *J* = 7.0 Hz, 6H, 2 × POCH_2_C*H*_3_) ppm; ^13^C NMR (151 MHz, CD_3_OD): *δ* = 167.34, 160.31, 154.19, 150.17, 143.87, 123.32, 61.90 (d, *J* = 6.7 Hz, POC), 45.15, 39.41 (d, *J* = 19.6 Hz, PCC*C*), 26.61, 22.11 (d, *J* = 4.9 Hz, PC*C*), 21.98 (d, *J* = 142.1 Hz, PC), 15.30 (d, *J* = 6.0 Hz, POC*C*) ppm; ^31^P NMR (243 MHz, CD_3_OD): *δ* = 32.75 ppm.

##### *Diethyl 2*-*[2*-*(2*-*amino*-*6*-*chloro*-*9H*-*purin*-*9*-*yl)acetamido]ethoxy)methyl)phosphonate* (**24d**, C_14_H_22_ClN_6_O_5_P)

The crude product obtained from 0.200 g diethyl (2-aminoethoxy)methylphosphonate (**7d**, 0.947 mmol) and 0.216 g 2-(2-amino-6-chloropurin-9-yl)acetic acid (**16**, 0.947 mmol) in the presence of 0.182 g EDC × HCl (0.947 mmol) and 0.132 cm^3^ TEA (0.947 mmol) according to the general procedure C was chromatographed with chloroform–methanol mixtures (50:1, 30:1 v/v) and the selected fractions were crystallized from an ethyl acetate–hexane mixture to give pure compound **24d** (0.048 g, 15% yield) as a white solid. M.p.: 91–93 °C; IR (KBr): $\bar{\nu }$ = 3430, 3335, 3220, 3095, 2981, 2938, 1650, 1611, 1245, 1024, 910 cm^−1^; ^1^H NMR (200 MHz, CD_3_OD): *δ* = 8.10 (s, 1H), 4.92 (s, 2H, C(O)CH_2_; superimposed in the HOD signal), 4.28–4.13 (m, 4H, 2 × POC*H*_2_CH_3_), 3.92 (d, *J* = 8.6 Hz, 2H, PCH_2_O), 3.70 (t, *J* = 5.3 Hz, 2H, PCH_2_OC*H*_2_CH_2_), 3.47 (t, *J* = 5.3 Hz, 2H, PCOCH_2_C*H*_2_), 1.37 (t, *J* = 7.1 Hz, 6H, 2 × POCH_2_C*H*_3_) ppm; ^13^C NMR (151 MHz, CD_3_OD): *δ* = 167.33, 160.30, 154.21, 150.14, 143.92, 123.25, 71.42 (d, *J* = 12.1 Hz, PCO*C*), 64.18 (d, *J* = 167.6 Hz, PC), 62.77 (d, *J* = 6.6 Hz, PO*C*), 44.96, 39.04, 15.36 (d, *J* = 5.5 Hz, POC*C*) ppm; ^31^P NMR (81 MHz, CD_3_OD): *δ* = 23.11 ppm.

##### Synthesis of the guanine derivatives **25a**–**25d** (general procedure)

A mixture of phosphonates **24a**–**24d** (1.00 mmol) and 8 cm^3^ of a 75% aqueous trifluoroacetic acid was stirred at room temperature for 48 h, concentrated in vacuo and co-evaporated with water (3 × 5 cm^3^), ethanol (3 × 5 cm^3^), and chloroform (3 × 5 cm^3^). The residue was crystallized from the appropriate solvent.

##### *Diethyl [2*-*(2*-*amino*-*1,6*-*dihydro*-*6*-*oxopurin*-*9*-*yl)acetamido]methylphosphonate* (**25a**, C_12_H_19_N_6_O_5_P × 2.5H_2_O)

From 0.112 g of compound **24a** (0.297 mmol) the phosphonate **25a** (0.087 g, 81%) was obtained as a white solid after crystallization from a methanol–diethyl ether mixture. M.p.: 165 °C (decomposition); IR (KBr): $\bar{\nu }$ = 3425, 2988, 2928, 2854, 2732, 1697, 1214, 1026 cm^−1^; ^1^H NMR (600 MHz, DMSO-*d*_6_): *δ* = 10.63 (s, 1H, NH), 8.61 (t, *J* = 5.9 Hz, 1H, C(O)N*H*CH_2_), 7.73 (s, 1H), 6.47 (s, 2H, NH_2_), 4.72 (s, 2H, C(O)CH_2_), 4.06–3.98 (m, 4H, 2 × POC*H*_2_CH_3_), 3.60 (dd, *J* = 11.8 Hz, *J* = 5.9 Hz, 2H, PCH_2_), 1.24 (t, *J* = 7.0 Hz, 6H, 2 × POCH_2_C*H*_3_) ppm; ^13^C NMR (151 MHz, DMSO-*d*_6_): *δ* = 166.96 (d, *J* = 4.5 Hz), 156.98, 154.22, 151.84, 138.76, 115.84, 62.35 (d, *J* = 6.0 Hz, POC), 45.24, 34.69 (d, *J* = 154.0 Hz, PC), 16.70 (d, *J* = 6.0 Hz, POC*C*) ppm; ^31^P NMR (243 MHz, DMSO-*d*_6_): *δ* = 22.46 ppm.

##### *Diethyl 2*-*[2*-*(2*-*amino*-*1,6*-*dihydro*-*6*-*oxopurin*-*9*-*yl)acetamido]ethylphosphonate* (**25b**, C_13_H_21_N_6_O_5_P × H_2_O)

From 0.127 g of compound **24b** (0.325 mmol) the phosphonate **25a** (0.109 g, 90%) was obtained as a white solid after crystallization from a methanol–diethyl ether mixture. M.p.: 175 °C (decomposition); IR (KBr): $\bar{\nu }$ = 3383, 2925, 2854, 1688, 1606, 1220, 1026 cm^−1^; ^1^H NMR (600 MHz, DMSO-*d*_6_): *δ* = 10.73 (s, 1H, NH), 8.35 (t, *J* = 5.5 Hz, 1H, C(O)N*H*CH_2_), 7.73 (s, 1H), 6.58 (s, 2H, NH_2_), 4.63 (s, 2H, C(O)CH_2_), 4.03–3.95 (m, 4H, 2 × POC*H*_2_CH_3_), 3.30-3.24 (m, 2H, PCH_2_C*H*_2_), 1.98–1.91 (m, 2H, 2H, PC*H*_2_), 1.24 (t, *J* = 7.0 Hz, 6H, 2 × POCH_2_C*H*_3_) ppm; ^13^C NMR (151 MHz, DMSO-*d*_6_): *δ* = 166.78, 157.00, 154.31, 151.82, 138.68, 116.03, 61.62 (d, *J* = 4.5 Hz, POC), 45.45, 33.77, 25.72 (d, *J* = 136.2 Hz, PC), 16.71 (d, *J* = 5.7 Hz, POC*C*) ppm; ^31^P NMR (81 MHz, DMSO-*d*_6_): *δ* = 30.12 ppm.

##### *Diethyl 3*-*[2*-*(2*-*amino*-*1,6*-*dihydro*-*6*-*oxopurin*-*9*-*yl)acetamido]propylphosphonate* (**25c**, C_14_H_23_N_6_O_5_P × 2H_2_O)

From 0.050 g of compound **24c** (0.12 mmol) the phosphonate **25c** (0.042 g, 87%) was obtained as a white solid after crystallization from a methanol–diethyl ether–petroleum ether mixture. M.p.: 155 °C (decomposition); IR (KBr): $\bar{\nu }$ = 3385, 3277, 2928, 2864, 1689, 1609, 1228, 1020 cm^−1^; ^1^H NMR (600 MHz, DMSO-*d*_6_): *δ* = 10.91 (s, 1H, NH), 8.29 (t, *J* = 5.5 Hz, 1H, C(O)N*H*CH_2_), 8.06 (s, 1H), 6.68 (s, 2H, NH_2_), 4.70 (s, 2H, C(O)CH_2_), 4.04-3.95 (m, 4H, 2 × POC*H*_2_CH_3_), 3.33 (dd, *J* = 12.7 Hz, *J* = 6.5 Hz, 2H, PCH_2_CH_2_C*H*_2_), 1.79–1.70 (m, 2H, PCH_2_C*H*_2_), 1.67–1.58 (m, 2H, PC*H*_2_CH_2_), 1.24 (t, *J* = 7.0 Hz, 6H, 2 × POCH_2_C*H*_3_) ppm; ^13^C NMR (151 MHz, DMSO-*d*_6_): *δ* = 166.36, 156.24, 154.69, 151.53, 138.77, 113.98, 61.42 (d, *J* = 6.4 Hz, POC), 45.74, 39.62 (d, *J* = 18.3 Hz, PCC*C*), 22.80 (d, *J* = 4.5 Hz, PC*C*), 22.65 (d, *J* = 140.4 Hz, PC), 16.74 (d, *J* = 4.5 Hz, POC*C*) ppm; ^31^P NMR (243 MHz, DMSO-*d*_6_): *δ* = 31.64 ppm.

##### *Diethyl 2*-*[2*-*(2*-*amino*-*1,6*-*dihydro*-*6*-*oxopurin*-*9*-*yl)acetamido]ethoxy)methyl)phosphonate* (**25d**, C_14_H_23_N_6_O_6_P × H_2_O)

From 0.012 g of compound **24d** (0.029 mmol) the phosphonate **25d** (0.010 g, 83%) was obtained as a yellowish oil. IR (film): $\bar{\nu }$ = 3378, 3288, 2938, 2881, 1690, 1611, 1225, 1018 cm^−1^; ^1^H NMR (600 MHz, DMSO-*d*_6_): *δ* = 10.64 (s, 1H), 8.28 (t, *J* = 5.6 Hz, 1H), 7.80 (brs, 1H), 6.50 (s, 2H), 4.66 (s, 2H, C(O)CH_2_), 4.08–4.03 (m, 4H, 2 × POC*H*_2_CH_3_), 3.83 (d, *J* = 8.0 Hz, 2H, PCH_2_O), 3.56 (t, *J* = 5.7 Hz, 2H, PCH_2_OC*H*_2_CH_2_), 3.27 (q, *J* = 5.7 Hz, 2H, PCOCH_2_C*H*_2_), 1.25 (t, *J* = 7.0 Hz, 6H, 2 × POCH_2_C*H*_3_) ppm; ^13^C NMR (151 MHz, DMSO-*d*_6_): *δ* = 166.87, 156.96, 154.28, 150.14, 138.78, 115.52, 71.44 (d, *J* = 11.0 Hz, PCO*C*), 64.50 (d, *J* = 162.9 Hz, PC), 62.23 (d, *J* = 6.3 Hz, PO*C*), 45.44, 39.02, 16.75 (d, *J* = 5.2 Hz, POC*C*) ppm; ^31^P NMR (243 MHz, DMSO-*d*_6_): *δ* = 21.45 ppm.

### Antiviral activity assays

The compounds were evaluated against the following viruses: herpes simplex virus type 1 (HSV-1) strain KOS, herpes simplex virus-2 strain G, thymidine kinase-deficient (TK^−^) HSV-1 KOS strain resistant to ACV (ACV^r^), adenovirus-2, vesicular stomatitis virus, varicella-zoster virus (VZV) strain Oka, TK^−^ VZV strain 07-1, human cytomegalovirus (HCMV) strains AD-169 and Davis, feline herpes virus (FHV), vaccinia virus Lederle strain, respiratory syncytial virus (RSV) strain Long, vesicular stomatitis virus (VSV), feline coronovirus (FIPV), Coxsackie B4, Parainfluenza 3, Influenza virus A (subtypes H1N1, H3N2), influenza virus B, Reovirus-1, Sindbis, Reovirus-1, Punta Toro, yellow fever virus. The antiviral, other than anti-HIV, assays were based on inhibition of virus-induced cytopathicity or plaque formation in human embryonic lung (HEL) fibroblasts, African green monkey cells (Vero), human epithelial cells (HeLa), Crandell–Rees feline kidney cells (CRFK) or Madin–Darby canine kidney cells (MDCK). Confluent cell cultures in microtiter 96-well plates were inoculated with 100 CCID_50_ of virus (1 CCID_50_ being the virus dose to infect 50% of the cell cultures) or with 20 plaque forming units (PFU) (VZV) in the presence of varying concentrations of the test compounds. Viral cytopathicity or plaque formation was recorded as soon as it reached completion in the control virus-infected cell cultures that were not treated with the test compounds. Antiviral activity was expressed as the EC_50_ or compound concentration required to reduce virus-induced cytopathogenicity or viral plaque formation by 50%. Cytotoxicity of the test compounds was expressed as the minimum cytotoxic concentration (MCC) or the compound concentration that caused a microscopically detectable alteration of cell morphology.

### Cytostatic activity assays

All assays were performed in 96-well microtiter plates. To each well were added (5–7.5) × 10^4^ tumor cells and a given amount of the test compound. The cells were allowed to proliferate for 48 h (murine leukemia L1210 cells) or 72 h (human lymphocytic CEM and human cervix carcinoma HeLa cells) at 37 °C in a humidified CO_2_-controlled atmosphere. At the end of the incubation period, the cells were counted in a Coulter counter. The IC_50_ (50% inhibitory concentration) was defined as the concentration of the compound that inhibited cell proliferation by 50%.
